# High-resolution computed tomography findings in eight patients with hantavirus pulmonary syndrome

**DOI:** 10.1590/0100-3984.2016.0093

**Published:** 2017

**Authors:** Diego de Lacerda Barbosa, Bruno Hochhegger, Arthur Soares Souza Jr., Gláucia Zanetti, Dante Luiz Escuissato, Gustavo de Souza Portes Meirelles, Marcelo Buarque de Gusmão Funari, Edson Marchiori

**Affiliations:** 1 MD, Universidade Federal do Rio de Janeiro (UFRJ), Rio de Janeiro, RJ, Brazil.; 2 MD, PhD, Santa Casa de Porto Alegre, Porto Alegre, RS, Brazil.; 3 MD, PhD, Faculdade de Medicina de São José do Rio Preto (Famerp) and Ultra X, São José do Rio Preto, SP, Brazil.; 4 MD, PhD, Universidade Federal do Rio de Janeiro (UFRJ), Rio de Janeiro, RJ, Brazil.; 5 MD, PhD, Universidade Federal do Paraná (UFPR), Curitiba, PR, Brazil.; 6 MD, PhD, Universidade Federal de São Paulo (Unifesp) and Grupo Fleury, São Paulo, SP, Brazil.; 7 MD, PhD, Faculdade de Medicina da Universidade de São Paulo (FMUSP), São Paulo, SP, Brazil.

**Keywords:** Hantavirus, Computed tomography, Lung infections

## Abstract

**Objective:**

The purpose of this study was to describe the high-resolution computed tomography (HRCT) findings in patients with hantavirus pulmonary syndrome (HPS).

**Materials and Methods:**

We retrospectively reviewed HRCT findings from eight cases of HPS. All patients were men, aged 19-70 (mean, 41.7) years. Diagnoses were established by serological test (enzyme-linked immunosorbent assay) in all patients. Two chest radiologists analyzed the images and reached decisions by consensus.

**Results:**

The predominant HRCT findings were ground-glass opacities (GGOs) and smooth inter- and intralobular septal thickening, found in all eight cases; however, the crazy-paving pattern was found in only three cases. Pleural effusion and peribronchovascular thickening were observed in five patients. The abnormalities were bilateral in all patients.

**Conclusion:**

The predominant HRCT findings in patients with HPS were GGOs and smooth inter- and intralobular septal thickening, which probably correlate with the histopathologic findings of pulmonary edema.

## Abstract (pt)

**Objetivo:**

Descrever os achados na tomografia computadorizada de alta resolução (TCAR) de pacientes com síndrome pulmonar do hantavírus.

**Materiais e Métodos:**

Revisamos, retrospectivamente, os achados tomográficos de oito pacientes portadores da síndrome pulmonar do hantavírus, todos do sexo masculino e com idade de 19 a 70 anos (média de 41,7 anos). O diagnóstico foi estabelecido por teste sorológico de ensaio de imunoabsorção enzimática em todos os pacientes. Dois radiologistas de tórax analisaram as imagens e decidiram em consenso.

**Resultados:**

Os achados predominantes na TCAR foram opacidades em vidro fosco e espessamento septal inter e intralobular, observados nos oito pacientes; porém, padrão de pavimentação em mosaico foi encontrado em apenas três deles. Derrame pleural e espessamento peribroncovascular foram observados em cinco pacientes. As anormalidades foram bilaterais em todos os pacientes.

**Conclusão:**

Opacidades em vidro fosco e espessamento septal inter e intralobular foram os achados predominantes na TCAR nos pacientes com síndrome pulmonar do hantavírus, o que, provavelmente, se correlaciona com achados histopatológicos de edema pulmonar.

## INTRODUCTION

Hantavirus pulmonary syndrome (HPS) is an emerging zoonotic disease caused by hantavirus, a RNA virus belonging to the family Bunyaviridae. In the Americas, natural reservoirs of the virus are rodents of the subfamily Sigmodontinae^(1-3)^. The genus *Hantavirus* consists of several viruses classified into two groups, each associated with a different clinical syndrome: the Old World hantaviruses, which cause hemorrhagic fever with renal syndrome or nephropathia epidemica; and the New World hantaviruses, related to HPS^(4-6)^.

Human infection occurs after inhalation of aerosolized rodent excreta, which enables the virus to infect the respiratory epithelium in the lower airways. In HPS, the virus disseminates through the respiratory endothelium, starting a pro-inflammatory cascade and an uncontrolled immune response that result in the destruction of respiratory epithelium, microvascular leakage, and pulmonary edema, affecting primarily the peribronchovascular portions of the lobules^(7)^. The clinical presentation of HPS is usually nonspecific, with flulike manifestations. A cough (initially nonproductive) typically signals the transition to the cardiopulmonary phase, in which a fulminant capillary leak syndrome may lead to rapidly progressive pulmonary edema and shock^(8)^.

Although a presumptive diagnosis of HPS can be made based on the patient's history and clinical and radiologic findings, the final diagnosis is usually made by serology [enzymelinked immunosorbent assay (ELISA)], which identifies specific antibodies of the immunoglobulin (Ig) M and IgG classes. Reverse-transcription and polymerase chain reaction (PCR) techniques can also be used to identify the virus^(9)^. Radiographically, both syndromes (hemorrhagic fever with renal syndrome and HPS) can show pulmonary involvement, but findings are more evident in HPS, which presents as interstitial edema with rapid progression to airspace disease.^(10)^

Although the high-resolution computed tomography (HRCT) findings of Old World hantaviruses have been well described, only three isolated case reports^(8,11,12)^ have described the tomographic aspects of HPS. To our knowledge, no study has examined HRCT findings in a series of patients with HPS. Thus, limited information is available about the CT features of this form of the disease. The purpose of this study was to describe HRCT findings in a series of eight patients with confirmed HPS.

## MATERIALS AND METHODS

Our institutional review board approved this study and waived the requirement for informed patient consent. All data used in this study were anonymized. We retrospectively reviewed the records of eight adult patients with confirmed hantavirus pulmonary infection. The patients were examined between 2003 and 2014 in six tertiary hospitals in Brazil. Diagnoses of HPS were based on medical histories, clinical courses, and imaging findings. However, serological tests (ELISA) were positive for *Hantavirus* in all patients.

Chest CT examinations were performed using a variety of helical scanners, as different hospitals were involved in this study. In initial examinations, HRCT images were obtained at full inspiration with 1-2-mm slice thicknesses at 5-10-mm intervals and reconstructed using a high-spatialfrequency reconstruction algorithm. The most recent CT examinations were performed using helical acquisition and reconstructed with 1-2.5-mm slice thicknesses and 1-2-mm intervals using a high-spatial-frequency reconstruction algorithm. The acquisition time was 0.5-1 s per rotation, peak voltage was 120 kVp, modulated tube current was 100-400 mA, pitch was 1, and matrix was 512 × 512 pixels.

Two chest radiologists with more than 15 years of experience independently reviewed the images using mediastinal (width, 350-450 HU; level, 10-20 HU) and lung (width, 1200-1600 HU; level, 2500-2700 HU) window settings. Final assessment was achieved by consensus. The radiologists were blinded to patient demographics, clinical data, and final diagnoses. The following HRCT patterns were assessed, following the definitions proposed in the Fleischner Society's Glossary of Terms for Thoracic Imaging^(13)^: ground-glass opacity (GGO), defined as hazy increased lung opacity with preservation of bronchial and vascular margins; smooth septal thickening, identified as thin linear opacities between secondary pulmonary lobules; peribronchovascular thickening, defined as thickening of the connective-tissue sheath enclosing the bronchi, pulmonary arteries, and lymphatic vessels; small nodules, defined as rounded or irregular opacities, well or poorly defined, with diameters of 2-10 mm; crazy-paving pattern, identified as thickened interlobular septa and intralobular lines superimposed on a background of GGO, resembling irregularly shaped paving stones; and consolidation, appearing as a homogeneous increase in pulmonary parenchymal attenuation that obscures the margins of vessels and airway walls.

The axial distribution of lesions in the lung parenchyma was classified as central, peripheral, or diffuse. The distribution was noted to be peripheral when abnormalities were predominant in the outer third of the lung periphery, and central when abnormalities were predominant in the inner two-thirds of the transverse plane. In the craniocaudal direction, lung zones were defined as upper, middle, and lower. The upper zone was defined as that above the level of the aortic arch, the middle zone was delineated between the aortic arch and carina, and the lower zone was defined as that below the level of the carina. Lymph node enlargement and pleural effusion were also assessed. In addition, the predominance of findings in one lung or symmetric involvement was recorded.

## RESULTS

**Figure 1:**
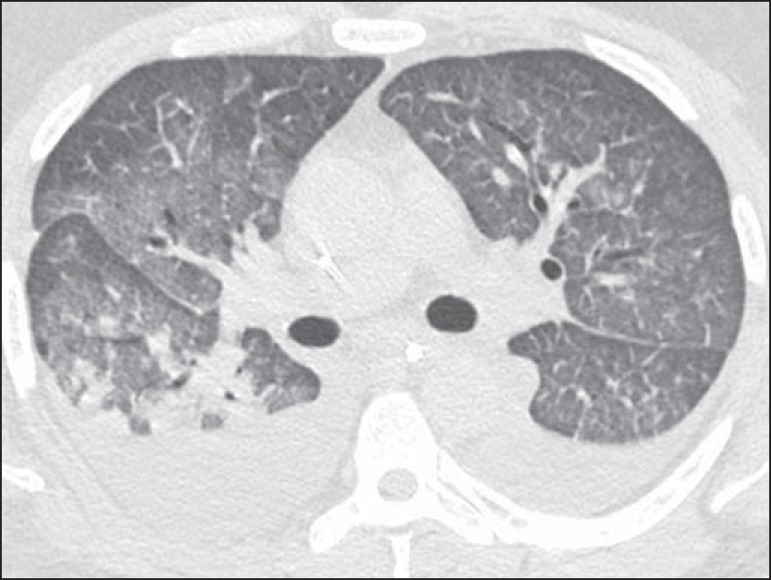
A 28-year-old man with fever and rapidly progressive dyspnea. High-resolution computed tomography with axial reconstruction shows bilateral ground-glass opacities. Note also bilateral pleural effusion.

**Figure 2:**
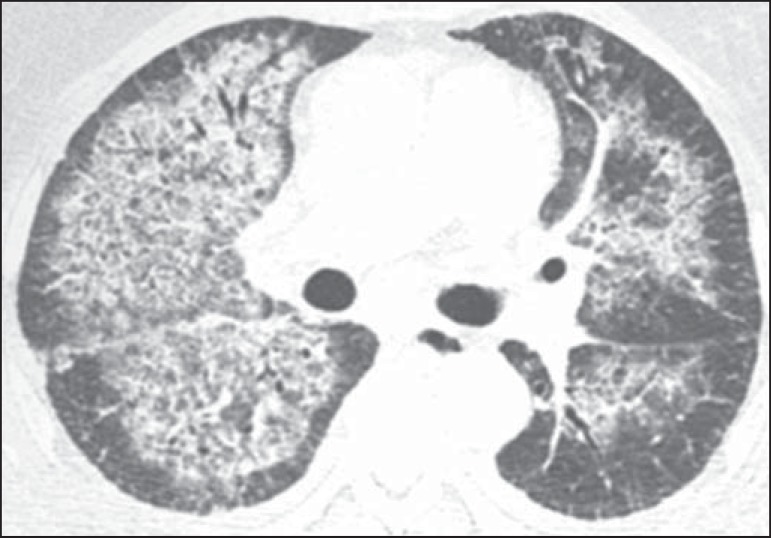
A 38-year-old man with dyspnea, fever, and myalgia, rapidly evolving to respiratory failure. Axial computed tomography demonstrates bilateral ground-glass opacities and smooth septal thickening with central distribution.

**Figure 3:**
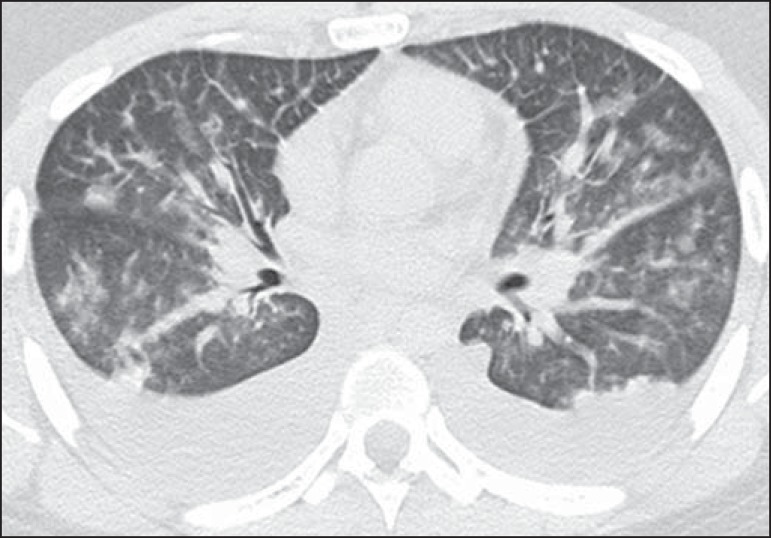
A 52-year-old man with fever and rapidly progressive dyspnea. Axial computed tomography demonstrates bilateral peribronchovascular thickening, ground-glass opacities, and bilateral pleural effusion.

**Figure 4:**
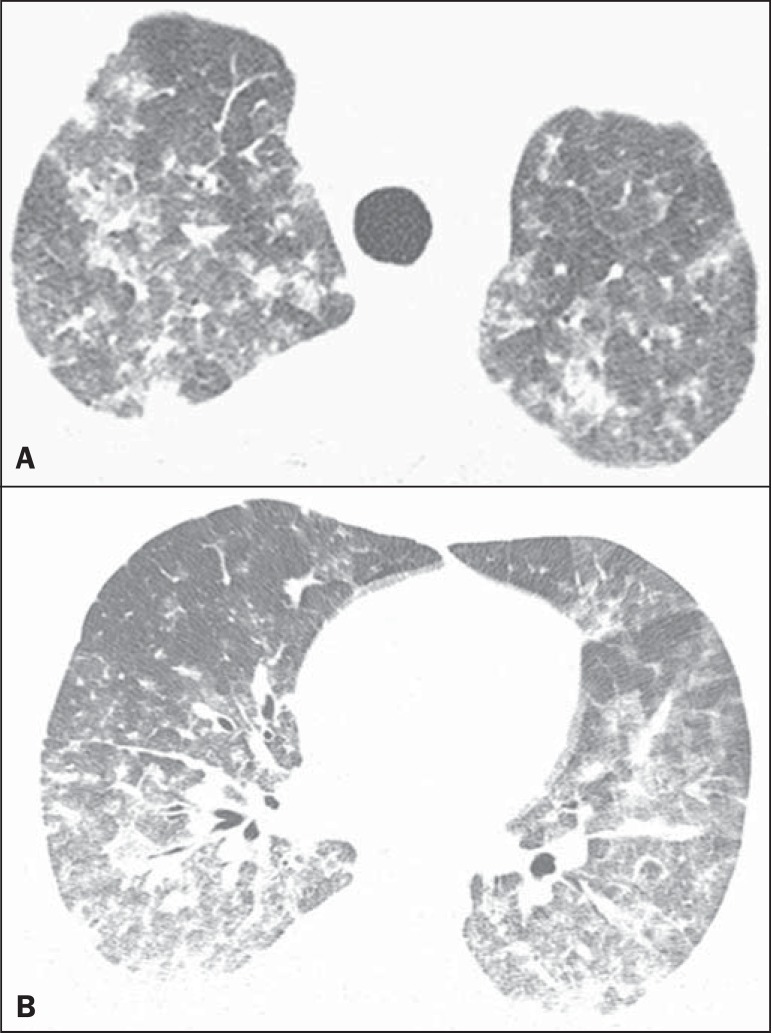
A 58-year-old man with fever and dyspnea. Axial high-resolution computed tomography images of the upper (**A**) and lower (**B**) lobes show bilateral areas of ground-glass opacity and small ill-defined nodules.

**Figure 5:**
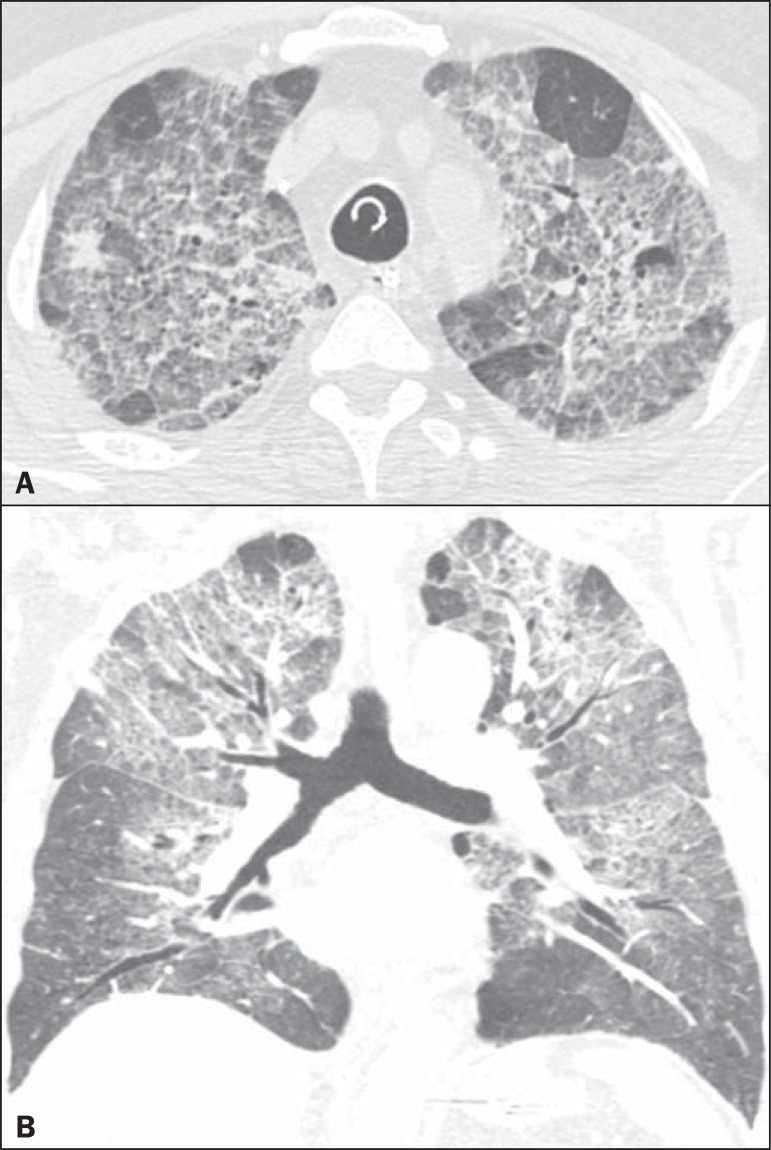
A 43-year-old man with dyspnea, dry cough, and fever. High-resolution computed tomography with axial (**A**) and coronal (**B**) reconstructions shows bilateral areas of ground-glass opacities associated with inter- and intralobular septal thickening ("crazy-paving" pattern).

The sample included eight men with a mean age of 41.7 (range, 19-70) years. All patients had reported risk factors related to the presence of mice in their homes or workplaces. Four patients died and four survived. The mean time from symptom onset to death was 4 (range, 2-6) days, the mean interval from the appearance of symptoms to discharge was 7.5 (range, 4-10) days, and the mean time from symptom onset to HRCT was 3 (range, 2-5) days. The main HRCT patterns (Table 1), found in all patients, were GGOs and smooth inter- and intralobular septal thickening (Figures 1 and 2). Pleural effusion and peribronchovascular thickening were observed in five cases (Figure 3). Four cases presented small nodules (Figure 4), and only one patient had an area of consolidation. The crazy-paving pattern was noted in three patients (Figure 5). The findings were distributed bilaterally and were diffuse in all cases; they were located in the central and peripheral zones of the lungs in seven patients and in the central region in one patient. The middle zone of the lungs was involved in all patients, the upper zone in five cases, and the lower zone in three cases. The abnormalities were asymmetric in seven patients and symmetric in one patient.

**Table 1:** High-resolution computed tomography findings in eight patients with hantavirus.

Finding	N	%
Ground-glass opacities	8	100%
Smooth septal thickening	8	100%
Pleural effusion	5	62.5%
Peribronchovascular thickening	5	62.5%
Small nodules	4	50%
Crazy-paving pattern	3	37.5%
Consolidation	1	12.5%

## DISCUSSION

Hantavirus can affect humans after inhalation of aerosolized virus particles from urine, saliva, or dried excreta of reservoir rodents^(11,14)^. Person-to-person transmission has been reported in a few cases, generally associated with a specific strain, the Andes virus^(8)^. In Brazil, 610 cases of HPS were reported from 1993 to November 2005, most occurring in the southern and southeastern regions of the country^(15)^. In these cases, hantavirus primarily affected young adult males and was related to occupational activities, tourism to rural areas, and flooding^(16)^. All patients in our sample were young males with positive epidemiological histories.

Endothelial damage, which causes an increase in capillary permeability, is the key pathophysiologic factor in HPS. This process occurs after hantaviruses infect the respiratory epithelium and disseminate via infected dendritic cells to monocytes and macrophages in the lymph nodes, and via the respiratory epithelium to the respiratory endothelium^(17)^. Infected endothelial cells produce proinflammatory chemokines attracting monocytes, macrophages, and T cells, producing additional proinflammatory molecules, such as tumor necrosis factor-α and reactive oxygen/nitrogen species, which are the main factors leading to vascular hyperpermeability and cardiovascular shock^(17)^. This process supports the long-standing belief that the induction of an uncontrolled immune response to hantavirus infection and the generation of a cytokine storm, rather than the viral infection per se, cause microvascular leakage and HPS.

The histopathologic course of the viral pneumonia associated with this infection begins with destruction and sloughing of respiratory cells, followed by bronchial and interstitial septal thickening due to edema and the activity of inflammatory cells^(7)^. This so-called interstitial pneumonitis is often patchy, affecting predominantly the peribronchovascular regions of the lobules. With more severe inflammation, the alveoli fill with inflammatory exudates, which may be hemorrhagic, and hyaline membranes may form^(7)^. Confirmation of the diagnosis of HPS requires virus-specific diagnostic tests, such as serological tests (ELISA), reverse transcription, and/or PCR^(6)^. ELISA results for all patients in our sample were positive for *Hantavirus*.

The Brazilian literature has reported the importance of imaging exams in the study of lung infections^(18-23)^. Radio-logically, chest X-ray, although nonspecific, is important to demonstrate pulmonary involvement in HPS; it is also essential for the monitoring of disease progression. A typical bilateral pattern of diffuse interstitial infiltration occurs early in more severe cases, at the onset of fever and dyspnea. These changes evolve rapidly along with the worsening of respiratory distress to confluence of lung infiltrates, resulting in alveolar consolidation. Pleural effusion is frequently reported, and often develops bilaterally. The infiltrates improve during the convalescent period, but may persist in the lung bases^(24,25)^.

Ketai et al.^(24)^ described a series of 16 patients with HPS seen in New Mexico. All patients presented with varying degrees of interstitial opacity on at least one chest radiograph taken during the course of the disease. All seven patients showing extensive airspace involvement on initial radiographs died. Deaths occurred within 48 hours of the development of extensive airspace disease. Of the nine surviving patients, the time to disease resolution ranged from 5 days to more than 3 weeks. In a review of chest X-ray findings for 20 patients with HPS, Boroja et al.^(25)^ identified two distinct patterns of presentation. The first, seen in 13/20 patients and associated with a fulminant clinical course, was characterized by rapid progression from bilateral interstitial changes to a bilateral interstitial pattern and airspace consolidation with pleural effusion. Six patients died within a few days. The second pattern, seen in 7/20 patients, was characterized by mild clinical symptoms and normal chest radiographic findings or the presence of minimal bilateral abnormalities. All of these patients recovered. In our sample, one patient who died showed a pattern of consolidation on the first chest X-ray examination. The other three demonstrated interstitial opacities, which evolved progressively to extensive air space consolidation. Two of the four patients who survived had normal chest radiographs upon admission. The others initially showed interstitial opacities, which regressed overtime. Pleural effusion was diagnosed on X-rays from three of the eight patients. The differences in findings may be explained by the modalities used for examination; CT is much more accurate than chest radiography, and parenchymal findings are easier to detect with HRCT^(26)^.

In our study, the main HRCT findings were GGOs and smooth interlobular septal thickening, observed in all patients. However, the crazy-paving pattern was observed in only three cases. Pleural effusion and peribronchovascular thickening were observed in five cases. Four patients had small nodules, and only one had foci of consolidation. Abnormalities were bilateral and diffuse in all patients. Few studies have described the HRCT manifestations of hantavirus infection. Most reported cases describe symptoms of infection with Old World hantaviruses (hemorrhagic fever with renal syndrome or nephropathia epidemica). Smooth septal and peribronchovascular thickening, central GGOs, the crazy-paving pattern, consolidation, and bilateral pleural effusion are the most commonly described patterns. Paakkala et al.^(26)^ reported that the main HRCT findings in 12 patients with nephropathia epidemica were atelectasis (11 patients), intralobular and interlobular septal thickening (7 patients), GGOs (4 patients), and bronchial wall thickening (2 patients). Pleural effusion was seen in 9 patients and was bilateral in 7. Hilar and mediastinal lymphadenopathy were seen in 3 patients. Rasmuson et al.^(27)^ showed that 14/27 patients with confirmed hantavirus infection had abnormal chest HRCT findings, with the most common patterns of pulmonary edema and pleural effusion found in 11 patients.

We found only three isolated case reports^(8,11,12)^ of CT findings associated with New World hantaviruses (HPS). Gasparetto et al.^(11)^ described the following HRCT findings of HPS in one patient: extensive bilateral GGOs, thickened interlobular septa, a few poorly defined small nodules, bronchial wall thickening, and small bilateral pleural effusions. Gonçalves et al.^(12)^ reported that HRCT demonstrated peribronchial cuffing, smooth septal thickening and central GGOs (resulting in a crazy-paving pattern), dependent areas of consolidation, and bilateral pleural effusions. Hamam et al.^(8)^ described bilateral alveolar consolidation with relative sparing of the peripheral lung fields, and small pleural effusions.

We found no reference to the crazy-paving pattern in case reports related to Old World hantaviruses. However, Gonçalves et al.^(12)^ described this pattern in a patient with HPS, and we identified it in three of eight patients. This finding was observed in two patients who survived, on the second and third days of disease, respectively, and on the third day of disease in a patient who died on the seventh day. Gonçalves et al.^(12)^ observed the pattern on the second day of evolution in a patient who died on the fifth day. Thus, the crazy paving pattern appears to have no relation to the severity of disease.

Lymphadenopathies have been described in patients infected with Old World hantaviruses. This finding was observed in 17% (4/24) of cases analyzed by Rasmuson et al.^(27)^, and in 23% (3/13) of cases reviewed by Paakkala et al.^(26)^. Linderholm et al.^(28)^ did not describe lymphadenopathy when reporting on 19 cases, and the finding was not observed in the three HPS cases described previously^(8,11,12)^. Similarly, none of our eight cases presented with lymphadenopathy. Five of our patients had pleural effusion, mild in two cases and moderate in three. Biochemical studies were not performed because the pleural effusion was bilateral and attributed to the underlying disease.

Although our sample was small, the patterns observed on chest X-rays by Boroja et al.^(25)^ appear to be reproducible on HRCT. In our study, the patient exhibiting a consolidation pattern died, but the other three patients who died showed only GGOs on HRCT. This presentation pattern is important because the patients were examined in the initial phase of the disease and did not undergo another examination due to rapid clinical worsening and morbid outcome. Yet, follow-up chest X-rays showed worsening of the findings, with air space disease. In the three previously described cases^(8,11,12)^, two patients with consolidation died, one after 10 hours and the other after 5 days. The patient who showed only GGOs had a good outcome and was discharged 10 days after symptom onset. Thus, the presence of extensive air space disease appears to be related to greater mortality. The differential diagnosis for this radiologic finding is broad, including pulmonary edema, pulmonary hemorrhage, and bacterial and viral pneumonia. The clinical history, in combination with laboratory and radiographic findings, may suggest the diagnosis of HPS. However, virus-specific diagnostic tests, such as serological tests, reverse transcription, or PCR, are required to confirm this diagnosis^(11)^. The differential diagnosis may be difficult with other viral diseases such as dengue fever^(29-31)^, Influenza A (H1N1)^(32-34)^, and even to bacterial infections, such as leptospirosis^(35-37)^. Our study has several limitations, including the observational retrospective design and the small sample. Although CT techniques varied widely because of the lengthy study period and differences in participating institutions' equipment, we do not believe that this variation impacted our results.

In conclusion, the predominant HRCT findings in patients with HPS were GGOs and smooth septal thickening. In the appropriate clinical setting, these findings have diagnostic value. Pleural effusion and peribronchovascular thickening are also frequent, but less characteristic, findings.
